# A sub-pharmacological test dose does not predict individual docetaxel exposure in prostate cancer patients

**DOI:** 10.1007/s00280-024-04684-2

**Published:** 2024-06-29

**Authors:** Marise R Heerma van Voss, Jessica Notohardjo, Joyce van Dodewaard-de Jong, Haiko J Bloemendal, Rob ter Heine

**Affiliations:** 1 Meander Medical Center, Department of Internal Medicine, Amersfoort, The Netherlands; 2 Department of Internal Medicine, Amsterdam University Medical Center, Amsterdam, The Netherlands; 3 Department of Medical Oncology, Research Institute for Medical Innovation, Radboudumc, Nijmegen, The Netherlands; 4 Department of Pharmacy, Research Institute for Medical Innovation, Radboudumc, Nijmegen, The Netherlands

**Keywords:** Docetaxel, Prostate cancer, Microdose, Pharmacokinetics

## Abstract

**Purpose:**

Docetaxel is a cytotoxic drug used for first-line treatment of various malignancies. It has a narrow therapeutic index and shows wide interpatient variability in clearance and toxicity. Tools for individual dose optimization are needed to maximize efficacy and avoid toxicity.

**Methods:**

We performed a proof-of-concept study (EudraCT 2016-003785-77) to evaluate whether pharmacokinetics after a sub-pharmacological test dose of 1000 µg docetaxel (millidose) could be used to predict therapeutic dose exposure. Thirty prostate cancer patients eligible for treatment with docetaxel as part of routine clinical care were included. An intravenous docetaxel millidose was administered 1–7 days prior to therapeutic docetaxel. After both doses plasma docetaxel concentrations were measured by ultra- high performance liquid chromatography-tandem mass spectrometry. The docetaxel clearance was estimated with non-linear mixed effects modeling.

**Results:**

Geometric mean docetaxel clearance was 57.9 L/h (GCV 78.6%) after admission of a millidose and 40.3 L/h (GCV 60.7%) after admission of a therapeutic dose. The millidose and therapeutic dose in a single patient were not significantly correlated (Spearman’s rho *R* = 0.02, *P* = 0.92).

**Conclusion:**

Docetaxel pharmacokinetics at milli- and therapeutic dose level showed insufficient correlation for individual dose optimization. However, the clearance of a docetaxel millidose and full dose are within the same order of magnitude. Therefore, docetaxel millidose pharmacokinetics could potentially facilitate prediction of docetaxel pharmacokinetics at a population level in situations where therapeutic dose levels are impractical, such as pharmacokinetic drug-drug interaction studies or pediatric studies.

**Supplementary Information:**

The online version contains supplementary material available at 10.1007/s00280-024-04684-2.

## Introduction

Docetaxel, a second-generation taxane, is one of the most widely used chemotherapeutic agents [1, 2] and is effective as monotherapy in a variety of tumor types, including breast, lung, and prostate cancer [2]. Currently, drug dosing is individualized based on body surface area (BSA) alone. However, body surface area is a poor predictor for systemic exposure [3], which shows a wide interpatient variability [4]. Since toxicity and efficacy of docetaxel are directly related to systemic exposure [5–7], this variability leads to unwanted outcomes like febrile neutropenia, which affects 15% of prostate cancer patients [8], or reduced efficacy due to subtherapeutic plasma levels. It is pivotal to develop a better dosing strategy for docetaxel from the first dose and onwards to reach an adequate systemic exposure ensuring maximal efficacy, while limiting toxicity.

Microdose phenotyping has been previously proposed as an attractive method to individualize dosing of anticancer drugs [9–11]. This strategy is appealing since it would also allow for correction of subtherapeutic dosing, in contrast to current clinical care in which dosing is only reduced in case of toxicity. In addition, it has the advantage of dose individualization from the first dose onwards, as opposed to pos-hoc adjustment with therapeutic drug monitoring. Docetaxel is dosed based on body surface area with therapeutic doses ranging from 75 to 100 mg/m^2^ on day one of a 21-day cycle. A microdose is defined as a dose of drug that is 1% of the pharmacologically active dose with a maximum of 100 µg [12]. Microdosing has an excellent track record of representing the pharmacokinetics of a drug at a therapeutic dose [13]. However, clinical implication of microdosing studies is often hindered by the lack of limited sampling strategies and absence of highly sensitive assays in the clinical setting. Therefore, we chose to use a subpharmacological (< 1% of a therapeutic dose) test dose of 1000 µg docetaxel, which we will call a “millidose” hereafter. We aimed to assess the potential of millidose pharmacokinetics to predict therapeutic docetaxel dose pharmacokinetics.

## Materials and methods

All patients who received docetaxel as part of routine care (for breast, prostate or non-small cell lung cancer) were eligible for participation in the Microdoce study. Thirty patients with prostate cancer treated with docetaxel 75 mg/m^2^ as part of routine clinical care in a general hospital between 2017 and 2020 were included. Written informed consent was retrieved from all patients. This study was conducted in accordance with the principles of the Declaration of Helsinki and was approved by the Medical research Ethical Committees United (MEC-U) in the Netherlands and was registered at clinicaltrials.eu (2016-003785-77).

Study subjects received an intravenous millidose of 1000 µg docetaxel (bolus), one to seven days prior to therapeutic docetaxel administration (60 min infusion). We verified the docetaxel concentration in the syringe before millidose administration. Blood samples were taken 0.25, 1, 2, 4, 6 and 8 h after the end of both the milli- and therapeutic dose. Samples were immediately centrifuged and stored at -80 °C until analysis. Plasma docetaxel concentrations were measured by high performance liquid chromatography-tandem mass spectrometry (LC-MS/MS; TSQ Altis, Triple Quadrupole Mass Spectrometer, Thermo Scientific). The lower limit of quantification was 0.01 µg/L. Bioanalytical inaccuracy and imprecision were less than 15% across the measured concentrations. Serum α-1-acidic glycoprotein (AAG) concentrations were measured on both days.

Dose and clearance are the only determinants for the area under the plasma concentration versus time curve (AUC) of a drug. In order to evaluate whether the therapeutic docetaxel dose AUC could be predicted using a millidose, we investigated the correlation between millidose and therapeutic dose docetaxel clearance. The majority of docetaxel (95%) is bound to plasma proteins. Since only the unbound docetaxel fraction is metabolized, the extent of plasma protein binding can have a substantial effect on docetaxel clearance. Interpatient variability in α-1-acidic glycoprotein (AAG) concentrations is considered a covariate for docetaxel protein binding [14]. Therefore, we measured AAG levels at the time of each docetaxel administration.

Pharmacokinetic analysis was performed by means of non-linear mixed effects modelling using the software package NONMEM V7.4 (ICON, Ireland) using the first order conditional estimation method with interaction (FOCE-I). In short, linear single and multiple compartment models were fitted to the obtained pharmacokinetic data in line with best practice [15]. Flow and volume parameters were allometrically scaled to a total body weight of 70 kg, with allometric coefficients of 0.75 and 1, as proposed earlier [16]. The datasets of the millidose and therapeutic dose pharmacokinetics were analyzed separately. Inter-individual variability was assumed to be log-normally distributed and for the residual error additive and proportional error models, as well as a combined additive and proportional error model were tested. Parameter precision was tested using the covariance option in nonmem. AAG was tested as a covariate for clearance. Individual empirical bayes estimates for clearance were obtained from the population pharmacokinetic analysis and these were correlated by means of Spearman’s rank correlation. Furthermore, the geometric mean ratio of millidose versus therapeutic docetaxel dose clearance was calculated.

## Results

The demographic data of the patient population are shown in Table 1. One patient was excluded during the study due to an allergic reaction to the docetaxel millidose. A two-compartment linear pharmacokinetic model best fitted the obtained pharmacokinetic curves for the millidose and the therapeutic dose of docetaxel. Inter-individual variability could be identified for clearance and the peripheral volume of distribution for the millidose dataset and for clearance and central volume of distribution for the therapeutic dose dataset. A proportional error model best described the residual error. During the blood sampling for the therapeutic dose, by mistake some samples were drawn during the infusion of docetaxel. As these concentrations drawn during infusion may still have been informative, yet less reliable, a separate proportional residual error was estimated for pharmacokinetic observations sampled during infusion to account for expected deviations. The difference in AAG serum concentration within individual patients on the day of the millidose and full dose docetaxel administration was minimal (0.03 g/L, IQR 0.01–0.05). AAG concentrations were not significantly correlated with clearance of docetaxel, and this covariate was, therefore, not retained in the developed models. A scatter plot of the empirical bayes estimates of docetaxel clearance versus AAG is provided in the supplemental material. Parameter estimates of the pharmacokinetic model are shown in Table 2. For further details on the pharmacokinetic models, including goodness-of-fit plots we refer to the supplemental material of this manuscript.

**Table 1:** Patient characteristics. * at time of millidose administration, ** at time of full dose docetaxel administration, *** between the day of millidose and full dose docetaxel administration, IQR = interquartile range, 95% CI = 95% confidence interval

Patient Characteristics		
Gender	Male (n)	29
Tumor type	Prostate (n)	29
Age (years)	Median (IQR)	72 (69–77)
BSA	Mean (95% CI)	2.02 (1.79–2.23)
Weight (kg)	Mean (95% CI)	85.5 (69.4-100.8)
Creatinine (umol/l)	Mean (95% CI)	90.9 (62.5-117.6)
Bilirubin (umol/l)	Mean (95% CI)	8.8 (5.2–13.9)
AAG day 1* (g/L)	Median (IQR)	0.73 (0.65-1.00)
AAG day 2** (g/L)	Median (IQR)	0.72 (0.63–0.88)
Intrapatient variability in AAG concentration*** (g/L)	Median (IQR)	0.03 (0.01–0.05)
Docetaxel millidose (mg)		1
Docetaxel full dose (mg)	Mean (95% CI)	151.2 (133.6–168.0)

**Table 2:** Parameter estimates of the pharmacokinetic model; RSE relative standard error of the estimates

	Estimate for the millidose (RSE)	Estimate for the therapeutic dose (RSE)
**Clearance (L/H)**	49.6 (27%)	34.6 (34%)
**Central volume of distribution (L)**	6,7 (62%)	8.00 (39%)
**Inter-compartmental clearance (L/H)**	39.2 (74%)	4.63 (20%)
**Peripheral volume of distribution (L)**	109 (50%)	22.2 (32%)
**Inter-individual variability in clearance (%)**	75.7 (15%)	61.7 (61%)
**Inter-individual variability in central volume of distribution (%)**	-	69.2 (87%)
**Inter-individual variability in peripheral volume of distribution (%)**	76.5 (27%)	-
**Residual error (%)**	37.0 (34%)	47.1 (21%)
**Residual error for samples drawn during infusion (%)**	-	98.8 (57%)

**Fig. 1:**
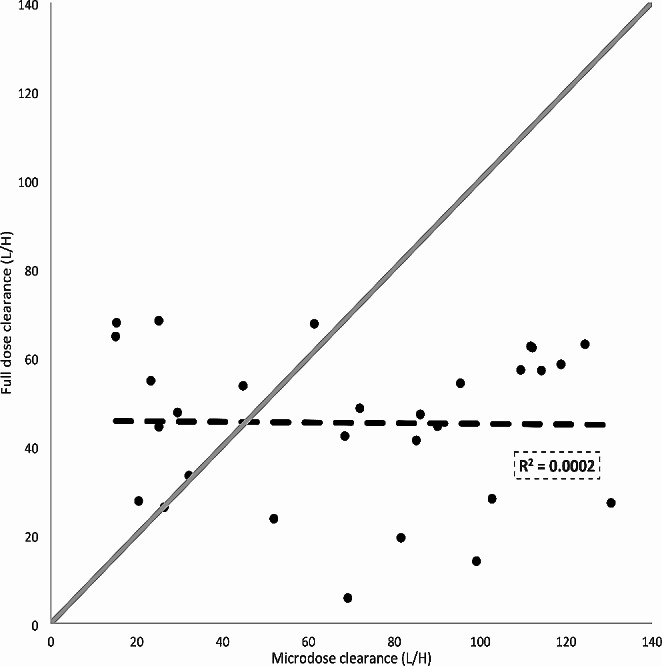
Correlation between full- and millidose docetaxel clearance. The black dashed line shows the linear regression line and the dotted grey line shows the line of unity

Figure 1 shows the empirical bayes estimates for therapeutic versus millidose docetaxel clearance.

Geometric mean docetaxel clearance was 57.9 L/h (geometric coefficient of variance; GCV 78.6%) after admission of a millidose and 40.3 L/h (GCV 60.7%) after admission of a therapeutic dose. The geometric mean ratio of millidose versus therapeutic dose docetaxel clearance was 1.44. Although the observed clearance on a population level was in the same order of magnitude, on an individual level the millidose and therapeutic dose in a single patient were not significantly correlated (Spearman’s rho *R* = 0.02, *P* = 0.92, Fig. 1).

## Discussion

In this study we show that there is no significant correlation between docetaxel millidose and therapeutic dose clearance. This finding is in line with a smaller study by Fujita et al., in which pharmacokinetics of a 100 µg docetaxel microdose were compared to those after full dose administration in nine patients [17]. Unfortunately, increasing the test dose to 1000 µg docetaxel and testing a larger cohort did not improve the observed correlation. A PET-imaging study using a [^11^C]docetaxel did show that therapeutic dose docetaxel volume of distribution and tumor uptake could be predicted using microdose data [18]. However, sampling in this study was limited to the first 80 min after infusion due to the short half-life of [^11^C] and therefore does not allow for comparison of terminal half-life and clearance.

Pharmacokinetic parameters were comparable to those described in literature. Docetaxel clearance was estimated around 37–45 L/h in other population pharmacokinetic studies [19, 20]. Interestingly, previous studies found docetaxel clearance to be 20–50% higher in patients with metastatic prostate cancer, as compared to patients with other solid tumors [20]. The geometric mean docetaxel clearance of 40.3 L/h and 57.9 L/h we found after full dose and millidose administration respectively, is similar to these previous reports in prostate cancer patients.

To investigate why we did not observe strong correlation between millidose and full dose docetaxel clearance, we verified the docetaxel concentration in the syringe prior to millidose administration. We hereby excluded solubility issues that could occur due to different concentrations of excipients.

Although the plasma AAG concentration is known to account for the largest variation in unbound fraction between patients [14], intrapatient AAG concentration was constant and correction for the unbound docetaxel fraction did not improve the observed correlation. An inverse correlation between docetaxel clearance and AAG concentration was reported at therapeutic dose levels [21], however a clear correlation at the millidose level was lacking in another study [17]. Potentially the contribution of AAG-binding to the total plasma protein binding of docetaxel is larger at therapeutic dose levels, due to saturation of binding to other proteins with higher binding constants and lower plasma concentrations (e.g. lipoproteins) [17].

Docetaxel clearance is largely hepatic and in addition to differences in the unbound fraction, interpatient variability in docetaxel exposure is largely attributable to CYP3A4 activity [14]. However, since CYP3A4 activity, is expected to be stable in individual patients, we do not expect CYP3A4 levels to explain why we did not observe a strong correlation between millidose and full dose clearance. Of note, several other studies found discrepancies between therapeutic and microdose PK-parameters, as has been discussed elsewhere [22].

Docetaxel dose optimization in individual patients remains an unresolved challenge. Although alternative methods like therapeutic drug monitoring and erythromycin breath tests have been proposed [23], these approaches have thus far failed to significantly reduce the interpatient variability in docetaxel exposure. Notably, chemotherapy-induced neutropenia has been linked to increased overall survival in non-small cell lung cancer patients [24]. Dose optimization algorithms based upon neutrophil counts after the first cycle of docetaxel have been suggested and deserve further clinical exploration [24]. Although docetaxel millidosing could not be used for dose optimization at the individual patient level, docetaxel clearance at milli- and therapeutic dose levels were within a two-fold range (geometric mean ratio 1.43) on a population level. This level of concordance is considered sufficient to facilitate pharmacokinetic studies that cannot be performed at therapeutic dose levels, like pharmacokinetic drug-drug interaction studies or pediatric studies [12].

## Electronic supplementary material

Below is the link to the electronic supplementary material.

Supplementary Material 1

## Data Availability

No datasets were generated or analysed during the current study.

## References

[1] Crown J, O’Leary M (2000) The taxanes: an update. Lancet 355(9210):1176–1178. 10.1016/S0140-6736(00)02074-2 10791395 10.1016/S0140-6736(00)02074-2

[2] Montero A, Fossella F, Hortobagyi G, Valero V (2005) Docetaxel for treatment of solid tumours: a systematic review of clinical data. Lancet Oncol 6(4):229–239. 10.1016/S1470-2045(05)70094-2 15811618 10.1016/S1470-2045(05)70094-2

[3] Felici A, Verweij J, Sparreboom A (2002) Dosing strategies for anticancer drugs: the good, the bad and body-surface area. Eur J Cancer 38(13):1677–1684. 10.1016/s0959-8049(02)00151-x 12175683 10.1016/s0959-8049(02)00151-x

[4] Hirth J, Watkins PB, Strawderman M, Schott A, Bruno R, Baker LH (2000) The effect of an individual’s cytochrome CYP3A4 activity on docetaxel clearance. Clin Cancer Res 6(4):1255–1258 10778948

[5] Puisset F, Alexandre J, Treluyer JM, Raoul V, Roche H, Goldwasser F, Chatelut E (2007) Clinical pharmacodynamic factors in docetaxel toxicity. Br J Cancer 97(3):290–296. 10.1038/sj.bjc.6603872 17595656 10.1038/sj.bjc.6603872 PMC2360335

[6] Bruno R, Hille D, Riva A, Vivier N, ten Bokkel Huinnink WW, van Oosterom AT, Kaye SB, Verweij J, Fossella FV, Valero V, Rigas JR, Seidman AD, Chevallier B, Fumoleau P, Burris HA, Ravdin PM, Sheiner LB (1998) Population pharmacokinetics/pharmacodynamics of docetaxel in phase II studies in patients with cancer. J Clin Oncology: Official J Am Soc Clin Oncol 16(1):187–196. 10.1200/JCO.1998.16.1.187 10.1200/JCO.1998.16.1.187 9440742

[7] Kushnir I, Mallick R, Ong M, Canil C, Bossé D, Koczka K, Reaume NM (2020) Docetaxel dose-intensity effect on overall survival in patients with metastatic castrate-sensitive prostate cancer. Cancer Chemother Pharmacol 85(5):863–868. 10.1007/s00280-020-04063-7 32240336 10.1007/s00280-020-04063-7

[8] James ND, Sydes MR, Clarke NW, Mason MD, Dearnaley DP, Spears MR, Ritchie AW, Parker CC, Russell JM, Attard G, de Bono J, Cross W, Jones RJ, Thalmann G, Amos C, Matheson D, Millman R, Alzouebi M, Beesley S, Birtle AJ, Brock S, Cathomas R, Chakraborti P, Chowdhury S, Cook A, Elliott T, Gale J, Gibbs S, Graham JD, Hetherington J, Hughes R, Laing R, McKinna F, McLaren DB, O’Sullivan JM, Parikh O, Peedell C, Protheroe A, Robinson AJ, Srihari N, Srinivasan R, Staffurth J, Sundar S, Tolan S, Tsang D, Wagstaff J, Parmar MK (2016) investigators S Addition of docetaxel, zoledronic acid, or both to first-line long-term hormone therapy in prostate cancer (STAMPEDE): survival results from an adaptive, multiarm, multistage, platform randomised controlled trial. Lancet 387 (10024):1163–1177. 10.1016/S0140-6736(15)01037-5 10.1016/S0140-6736(15)01037-5 PMC4800035 26719232

[9] Hohmann N, Haefeli WE, Mikus G (2015) Use of Microdose phenotyping to Individualise Dosing of patients. Clin Pharmacokinet 54(9):893–900. 10.1007/s40262-015-0278-y 25925712 10.1007/s40262-015-0278-y

[10] van der Heijden L, van Nuland M, Beijnen J, Huitema A, Dorlo T (2023) A naïve pooled data approach for extrapolation of phase 0 microdose trials to therapeutic dosing regimens. Clin Transl Sci 16(2):258–268. 10.1111/cts.13446 36419385 10.1111/cts.13446 PMC9926085

[11] Boosman RJ, de Rouw N, Huitema ADR, Burgers JA, ter Heine R (2023) Prediction of the pharmacokinetics of pemetrexed with a low test dose: a proof-of-concept study. Br J Clin Pharmacol 89(2):699–704. 10.1111/bcp.15520 36053283 10.1111/bcp.15520

[12] Lappin G, Noveck R, Burt T (2013) Microdosing and drug development: past, present and future. Expert Opin Drug Metab Toxicol 9(7):817–834. 10.1517/17425255.2013.786042 23550938 10.1517/17425255.2013.786042 PMC4532546

[13] Rowland M (2012) Microdosing: a critical assessment of human data. J Pharm Sci 101(11):4067–4074. 10.1002/jps.23290 22927093 10.1002/jps.23290

[14] Clarke SJ, Rivory LP (1999) Clinical pharmacokinetics of docetaxel. Clin Pharmacokinet 36(2):99–114. 10.2165/00003088-199936020-00002 10092957 10.2165/00003088-199936020-00002

[15] Byon W, Smith MK, Chan P, Tortorici MA, Riley S, Dai H, Dong J, Ruiz-Garcia A, Sweeney K, Cronenberger C (2013) Establishing best practices and guidance in population modeling: an experience with an internal population pharmacokinetic analysis guidance. CPT Pharmacometrics Syst Pharmacol 2(7):e51. 10.1038/psp.2013.26 23836283 10.1038/psp.2013.26 PMC6483270

[16] Anderson BJ, Holford NH (2008) Mechanism-based concepts of size and maturity in pharmacokinetics. Annu Rev Pharmacol Toxicol 48:303–332. 10.1146/annurev.pharmtox.48.113006.094708 17914927 10.1146/annurev.pharmtox.48.113006.094708

[17] Fujita K, Yoshino E, Kawara K, Maeda K, Kusuhara H, Sugiyama Y, Yokoyama T, Kaneta T, Ishida H, Sasaki Y (2015) A clinical pharmacokinetic microdosing study of docetaxel with Japanese patients with cancer. Cancer Chemother Pharmacol 76(4):793–801. 10.1007/s00280-015-2844-2 26297058 10.1007/s00280-015-2844-2

[18] van der Veldt AA, Lubberink M, Mathijssen RH, Loos WJ, Herder GJ, Greuter HN, Comans EF, Rutten HB, Eriksson J, Windhorst AD, Hendrikse NH, Postmus PE, Smit EF, Lammertsma AA (2013) Toward prediction of efficacy of chemotherapy: a proof of concept study in lung cancer patients using [(1)(1)C]docetaxel and positron emission tomography. Clin Cancer Res 19(15):4163–4173. 10.1158/1078-0432.CCR-12-3779 23620410 10.1158/1078-0432.CCR-12-3779

[19] Bruno R, Vivier N, Vergniol JC, De Phillips SL, Montay G, Sheiner LB (1996) A population pharmacokinetic model for docetaxel (Taxotere): model building and validation. J Pharmacokinet Biopharm 24(2):153–172. 10.1007/BF02353487 8875345 10.1007/BF02353487

[20] Crombag MBS, Dorlo TPC, van der Pan E, van Straten A, Bergman AM, van Erp NP, Beijnen JH, Huitema ADR (2019) Exposure to Docetaxel in the Elderly Patient Population: a Population Pharmacokinetic Study. Pharm Res 36(12):181. 10.1007/s11095-019-2706-4 31732882 10.1007/s11095-019-2706-4

[21] Minami H, Kawada K, Sasaki Y, Tahara M, Igarashi T, Itoh K, Fujii H, Saeki T, Ozawa K, Sato H (2009) Population pharmacokinetics of docetaxel in patients with hepatic dysfunction treated in an oncology practice. Cancer Sci 100(1):144–149. 10.1111/j.1349-7006.2009.00992.x 19018756 10.1111/j.1349-7006.2009.00992.x PMC11158642

[22] Bosgra S, Vlaming ML, Vaes WH (2016) To apply Microdosing or not? Recommendations to single out compounds with non-linear pharmacokinetics. Clin Pharmacokinet 55(1):1–15. 10.1007/s40262-015-0308-9 26242381 10.1007/s40262-015-0308-9

[23] Engels FK, Loos WJ, van der Bol JM, de Bruijn P, Mathijssen RH, Verweij J, Mathot RA (2011) Therapeutic drug monitoring for the individualization of docetaxel dosing: a randomized pharmacokinetic study. Clin Cancer Res 17(2):353–362. 10.1158/1078-0432.CCR-10-1636 21224369 10.1158/1078-0432.CCR-10-1636

[24] Lombard A, Mistry H, Aarons L, Ogungbenro K (2021) Dose individualisation in oncology using chemotherapy-induced neutropenia: example of docetaxel in non-small cell lung cancer patients. Br J Clin Pharmacol 87(4):2053–2063. 10.1111/bcp.14614 33075149 10.1111/bcp.14614

