# Supplementary material for: A sub-pharmacological test dose does not predict individual docetaxel exposure in prostate cancer patients
Source: Cancer Chemother Pharmacol. 2024 Jun 29;94(3):437–41. doi: 10.1007/s00280-024-04684-2 (PMC11420247; doi:10.1007/s00280-024-04684-2)
Supplement: Supplementary file 1 — Supplementary Material 1 [file 280_2024_4684_MOESM1_ESM.docx]

**Supplemental material – additional pharmacokinetic analysis results**

**A sub-pharmacological test dose does not predict individual docetaxel exposure in prostate cancer patients**

**Marise Heerma van Voss^1,2^*, Jessica Notohardjo^1,2^*, Joyce van Dodewaard-de Jong^1^, Haiko J Bloemendal^1,3^, Rob ter Heine^4^**

1. Meander Medical Center, department of internal medicine, Amersfoort, The Netherlands

2. Amsterdam University Medical Center, department of internal medicine, Amsterdam, The Netherlands

3. Radboudumc, Research Institute for Medical Innovation, department of Medical Oncology, Nijmegen, The Netherlands

4. Radboudumc, Research Institute for Medical Innovation, department of Pharmacy, Nijmegen, The Netherlands


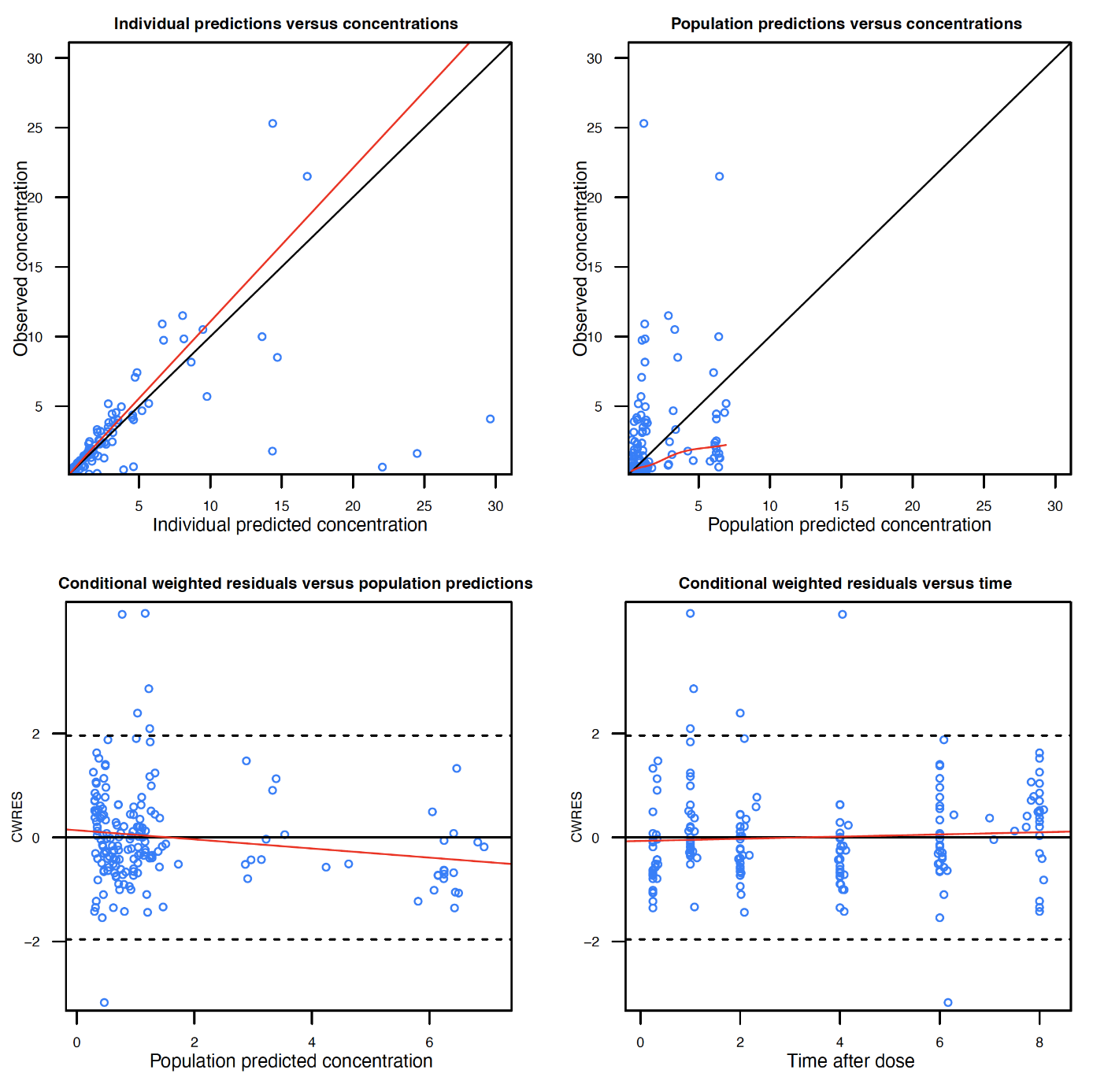
Goodness-of-fit plots for the millidose dataset

Concentrations are depicted in microgram/L units. Time units are in hours. The red line in each pane depicts the linear trendline.


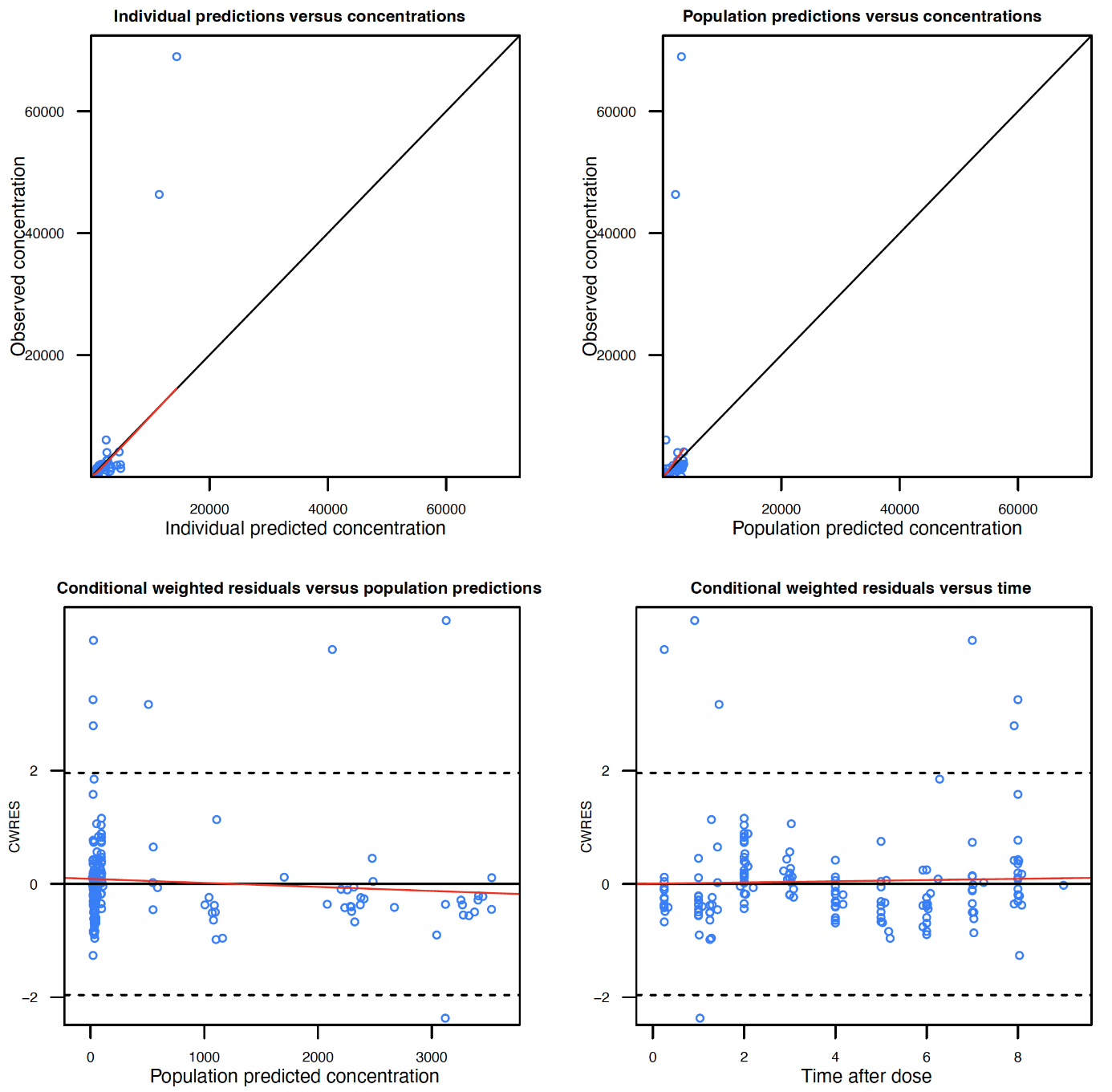
Goodness-of-fit plots for the therapeutic dataset

Concentrations are depicted in microgram/L units. Time units are in hours. The red line in each pane depicts the linear trendline.

Visual predictive check for the millidose dataset


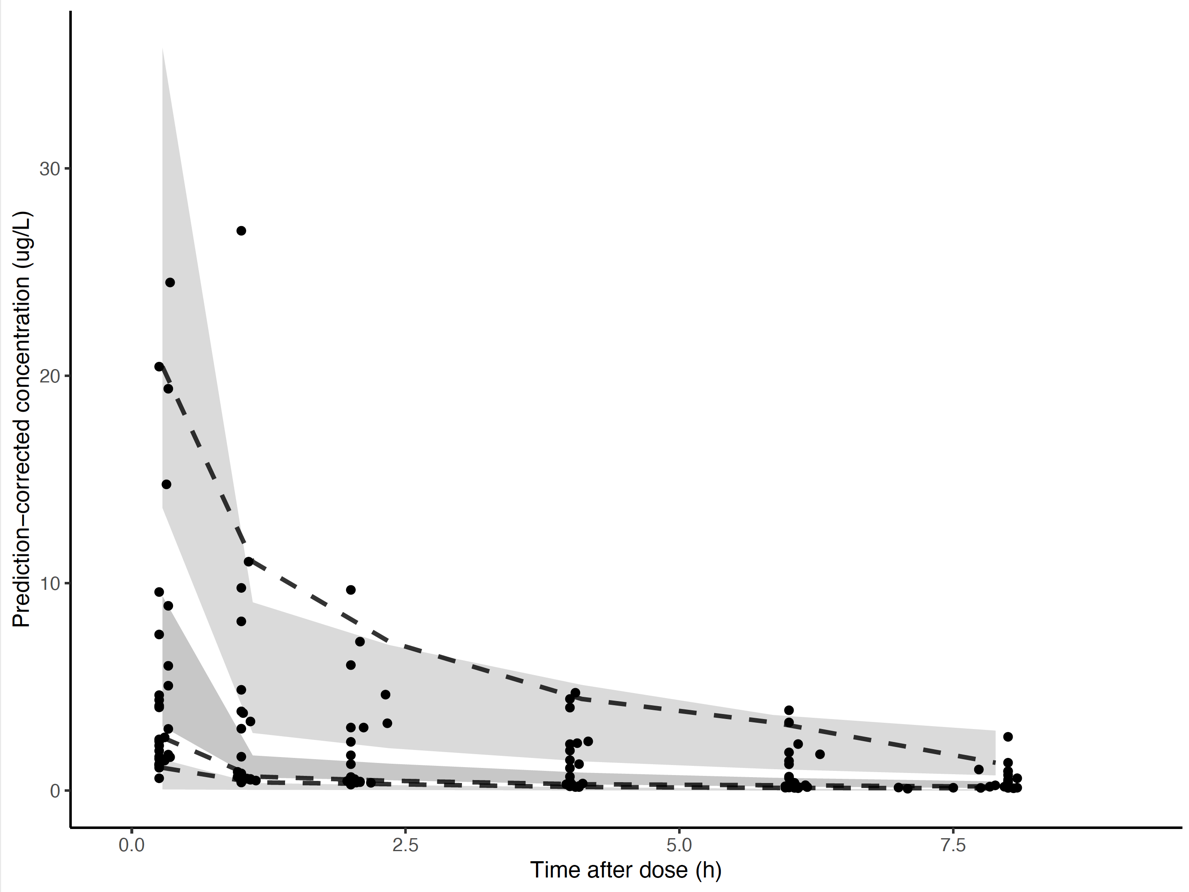


Visual predictive check for the therapeutic dose dataset


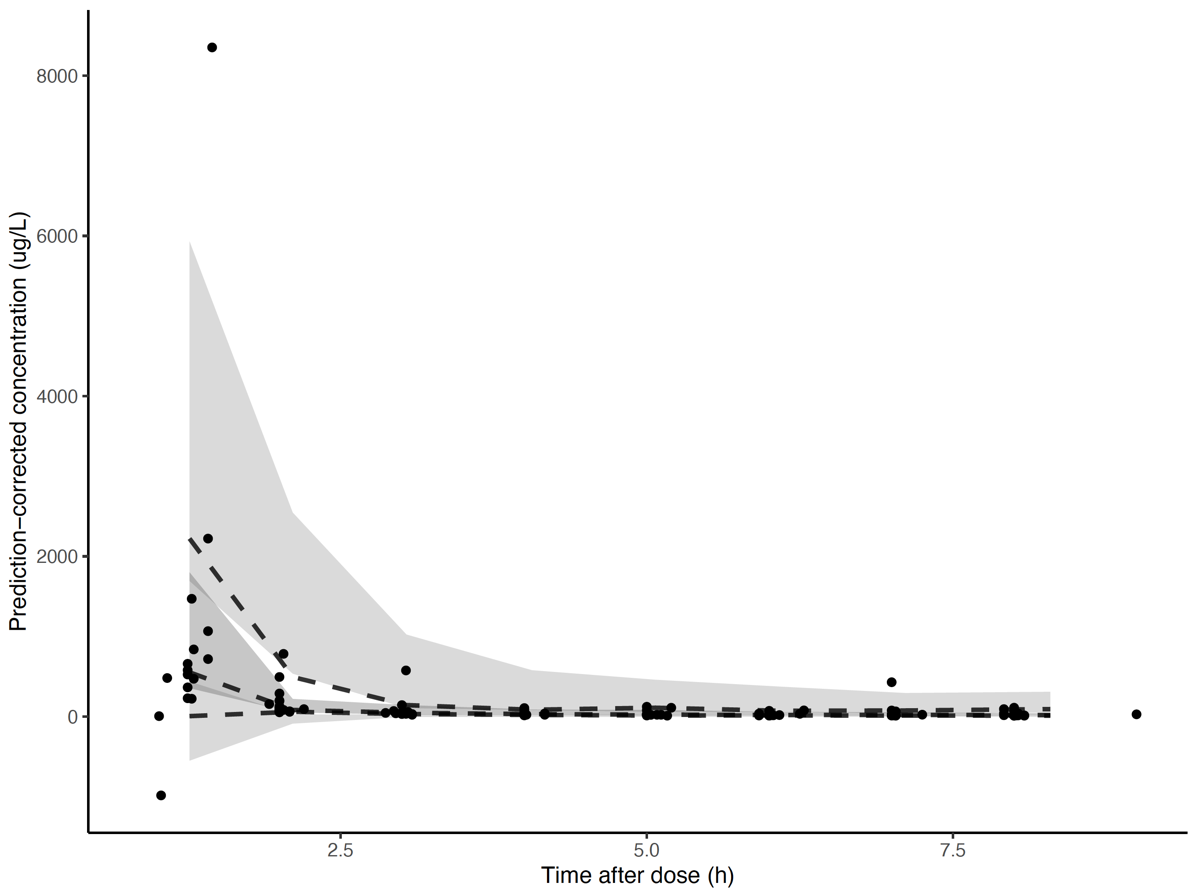


Docetaxel clearance versus AAG concentrations

The black and red dots represent the empirical bayes estimates for the docetaxel clearance versus AAG for the therapeutic and millidose, respectively.
